# Supplementary material for: High-resolution genomic history of early medieval Europe
Source: Nature. 2025 Jan 1;637(8044):118–26. doi: 10.1038/s41586-024-08275-2 (PMC11693606; doi:10.1038/s41586-024-08275-2)
Supplement: Supplementary file 1 — Mathematical derivation of the optimal Twigstats cut-off for a four-population f4-statistic f4(PO, P2, PX, P1), where PX is a mixture of P1 and P2. [file 41586_2024_8275_MOESM1_ESM.pdf]

nature portfolio

https://doi.org/10.1038/s41586-024-08275-2

Supplementary information

High-resolution genomic history of early medieval Europe

In the format provided by the
authors and unedited

Supplementary Note

Contents

1 Time-stratified *f*-statistics **1**

2 The optimal *Twigstats* cutoff **1**
2.1 Decomposing f4-statistics by how lineages coalesce back in time 1
2.2 Expected value of the f4-statistics 2
2.3 Derivation of the optimal *Twigstats* cutoff 3
2.4 Evaluating the theoretical z-score given a *Twigstats* cutoff 4
2.5 Evaluating the optimal *Twigstats* cutoff 5

1 Time-stratified *f*-statistics

Our *Twigstats* approach time-stratifies f-statistics by only considering lineages or mutations within a specified time period.

Specifically, our approach aims to boost statistical power of f-statistics for capturing recent demographic events by ascertaining recent genetic variation. Consider two populations that are recently separated. In such a case, looking backward in time, no coalescences should occur before these populations merge, and observing a lack of such recent coalescences is highly indicative of their reproductive isolation. However, since old coalescences are common, these may dominate the values of f-statistics or the variance of f-statistics across the genome, and reduce power to detect recent events. We therefore expect a statistical benefit to exclude old coalescence events that are less informative.

Computation of f_2-statistics from genealogies and date ascertained mutations is implemented in an R package (*Twigstats*), which is compatible with the admixtools R package (https://github.com/uqrmaie1/admixtools) [1]. The admixtools R package framework utilises the fact that f3 and f4 statistics can be calculated as functions of f2 statistics and allows us to easily access other well-used f-statistics-derived approaches, including *qpAdm*.

2 The optimal *Twigstats* cutoff

The time cutoff that minimises the standard error of computed f-statistics is a priori unknown but one can make an informed guess. It is useful to think of the best cutoff time when true underlying genealogies are known and f-statistics are calculated directly on these genealogies. As we will demonstrate here, the optimal *Twigstats* cutoff exceeds the split time of source groups involved in the admixture event by at most a factor of 1.4.

2.1 Decomposing f4-statistics by how lineages coalesce back in time

Let us consider the following scenario. We consider a group P_X resulting from a two-way admixture between source groups P_1 and P_2 some time t_a ago, with the two source groups separating at time $t_s > t_a$ ago. For simplicity, let us assume that there is an outgroup P_O, which is sufficiently removed such that it does not interfere with the optimal *Twigstats* cutoff.

Let us denote by τ the *Twigstats* cutoff time. Let us assume that there are L independent loci in the genome. We denote by a_P the frequency of alleles in each population P. We are interested in computing an f_4-statistic of the form

$$f_4^{(\tau)}(P_O, P_2, P_X, P_1) = \frac{1}{L}\sum_{\ell=1}^{L} E_{\ell,\tau}\left[(a_O - a_2)(a_X - a_1)|T_\ell\right], \tag{1}$$

which is negative if P_X carries ancestry from P_2 and zero if P_X is unadmixed and instead clusters with P_1 to the exclusion of P_2. The expectation in Eq. (1) is taken over branches in the marginal coalescent tree T_ℓ at locus ℓ assuming a constant mutation rate. Eq. (1) is therefore a random variable that depends on the realised marginal trees at L loci. We will decompose the $f_4^{(\tau)}$ statistic by considering different possible marginal trees.

We now sample one lineage from each of the four populations P_X, P_1, P_2, and P_O. Allele frequencies a_X, a_1, a_2, a_O can therefore only take values 0 or 1. Let us denote by $\{X \to 2\}$ and $\{X \to 1\}$ the events that the lineage in X migrates to population P_2 or P_1 back in time. These events occur with probability α and $1-\alpha$, respectively, corresponding to the admixture proportions. We note that migration does not imply coalescence and that a lineage that migrated to P_2 may not coalesce until the source split time t_s and can then coalesce with P_1 before coalescing with P_2. We assume a constant effective population size throughout, and set this without loss of generality to 1.

We can now decompose our $f_4^{(\tau)}$-statistic and obtain,

$$f_4^{(\tau)} = \mathbb{1}_{\{X\to 1\}} f_4^{(\tau)}(X \to 1) + \mathbb{1}_{\{X\to 2\}} f_4^{(\tau)}(X \to 2) \tag{2}$$

Let us further denote by $\{(X,1);t\}$, $\{(X,2);t\}$, and $\{(1,2);t\}$ the disjoint events that the first coalescence of these lineages occurs between the populations shown in brackets and at time t, ignoring for now which population the lineage in P_X migrated to. Each of these events, when realised, contribute a different amount to our f_4-statistic in Eq. (1).

The event $\{(X,1);t\}$ always contributes 0 to the $f_4^{(\tau)}$ statistic, the event $\{(X,2);t\}$ contributes a negative value that depends on time t, and $\{(1,2);t\}$ contributes a positive value that depends on time t. The exact value that is being contributed is computed by integrating over possible times when the coalesced lineages coalesces with the remaining third lineage, and is therefore a function of time t. We further note that because all effective population sizes are the same, and sample sizes in each population are identical (equalling 1), these events are equally likely if $t > t_s$, or in other words it does not matter whether the lineage of population X migrated to population 1 or 2.

We use these events to further decompose the $f_4^{(\tau)}$ statistics. Starting with $f_4^{(\tau)}(X \to 1)$, a coalescence $t < t_s$ is only possible with population 1 which contributes 0, and therefore, the only non-zero contributions arise when the first coalescence is $t > t_s$ and we obtain,

$$f_4^{(\tau)}(X \to 1) = \mathbb{1}_{\{(X,2);t>t_s\}} f_4^{(\tau)}((X,2);t > t_s) + \mathbb{1}_{\{(1,2);t>t_s\}} f_4^{(\tau)}((1,2);t > t_s). \tag{3}$$

Next, we can decompose $f_4^{(\tau)}(X \to 2)$. Here, a coalescence at times $t < t_s$ with population 2 contributes negatively, and so we obtain,

$$f_4^{(\tau)}(X \to 2) = \mathbb{1}_{\{(X,2);t\le t_s\}} f_4^{(\tau)}((X,2);t \le t_s) + \mathbb{1}_{\{(X,2);t>t_s\}} f_4^{(\tau)}((X,2);t > t_s) + \mathbb{1}_{\{(1,2);t>t_s\}} f_4^{(\tau)}((1,2);t > t_s). \tag{4}$$

By substituting Eqs. (3) and (4) in Eq. (2), we obtain

$$f_4^{(\tau)} = \mathbb{1}_{\{X\to 2\}} \mathbb{1}_{\{(X,2);t\le t_s\}} f_4^{(\tau)}((X,2);t \le t_s) + \mathbb{1}_{\{(X,2);t>t_s\}} f_4^{(\tau)}((X,2);t > t_s) + \mathbb{1}_{\{(1,2);t>t_s\}} f_4^{(\tau)}((1,2);t > t_s). \tag{5}$$

2.2 Expected value of the f4-statistics

We can now compute the expectation of Eq. (5). Since all coalescence rates are equal at $t > t_s$, we note that

$$f_4^{(\tau)}((X,2);t > t_s) = -f_4^{(\tau)}((1,2);t > t_s), \tag{6}$$

and because events $\{(X,2);t\}$ and $\{(1,2);t\}$ are equally likely, we get that the last two terms in Eq. (5) cancel out in expectation. The probability of the lineage in population X migrating to population 2 is *alpha* and the probability of it coalescing with the lineage in population 2 before t_s is given by $1-e^{-(t_s-t_a)}$ and we therefore obtain

$$E[f_4^{(\tau)}] = P(X \to 2) P\left((X,2); t \leq t_s\right) E[f_4^{(\tau)}((X,2); t \leq t_s)] \tag{7}$$

$$= \alpha \left(1 - e^{-(t_s - t_a)}\right) E[f_4^{(\tau)}((X,2); t \leq t_s)]. \tag{8}$$

In other words, terms that on-average have a non-zero contribution on the $f_4^{(\tau)}$ statistic arise only through the combination of events $\{X \to 2\}$ and $\{(X,2); t \leq t_s\}$ and all other terms, which correspond to cases where the first coalescence occurs at times $t > t_s$, purely contribute noise to the $f_4^{(\tau)}$ statistic. The term $E[f_4^{(\tau)}((X,2); t \leq t_s)]$ corresponds to the expected number of mutations observed on a branch where X and 2 coalesce.

This indicates a tradeoff: We expect a lower *Twigstats* cutoff to increase power because it reduces the chance of a coalesce $t > t_s$ from occurring, which only contribute noise. This is because lineages either coalesce before t_s or, with increasing chance, don't coalesce before the *Twigstats* cutoff. On the other hand, once a coalescence $t \leq t_s$ between X and 2 has occurred, which will have a non-zero contribution to the $f_4^{(\tau)}$-statistic that is maximally informative of the admixture, we now want to maximally count mutations on the resulting coalesced lineage, i.e. ideally count the entire branch until it coalesces with the remaining lineage originally in population 1. This is achieved by a larger cutoff time.

2.3 Derivation of the optimal *Twigstats* cutoff

Given this trade-off, there should be an optimal choice for the *Twigstats* cutoff. To find this optimal value, we are interested in maximising

$$\frac{E[f_4^{(\tau)}]}{\sqrt{Var[f_4^{(\tau)}]}}, \tag{9}$$

which can be interpreted as a z-score, or inverse coefficient of variation. It will suffice to compute these at a single locus and the genome-wide value is obtained by multiplying by $\sqrt{L}$, assuming we have L independent loci.

It will be convenient to square Eq. (9) and we obtain

$$\frac{E\left[f_4^{(\tau)}\right]^2}{Var\left[f_4^{(\tau)}\right]} = \frac{E\left[f_4^{(\tau)}\right]^2}{E\left[\left(f_4^{(\tau)}\right)^2\right] - E\left[f_4^{(\tau)}\right]^2} = \left(\frac{E\left[\left(f_4^{(\tau)}\right)^2\right]}{E\left[f_4^{(\tau)}\right]^2} - 1\right)^{-1}, \tag{10}$$

and we therefore need to compute the first two moments of our $f_4^{(\tau)}$-statistic. The first moment is given by Eq. (7). For the second moment, we use our decomposition of the $f_4^{(\tau)}$-statistic in Eq. (5). Since Eq. (5) is a sum of disjoint events, we can simply square each term. We note that the probability of an event $\{(X,2); t > t_s\}$ is given by $e^{-(t_s-t_a)}/3$, because the P_X lineage is not allowed to coalesce before time t_s with either P_1 or P_2 (depending on which population it migrated to) and then has to coalesce with P_2 which has a chance of 1/3. This probability is the same for

event $\{(1,2); t > t_s\}$. We therefore obtain

$$E\left[(f_4^{(\tau)})^2\right] = \alpha\left(1 - e^{-(t_s - t_a)}\right) E\left[\left(f_4^{(\tau)}((X,2); t \le t_s)\right)^2\right] \tag{11}$$

$$+ \frac{e^{-(t_s - t_a)}}{3}\left(E\left[\left(f_4^{(\tau)}((X,2); t > t_s)\right)^2\right] + E\left[\left(f_4^{(\tau)}((1,2); t > t_s)\right)^2\right]\right) \tag{12}$$

$$= \alpha\left(1 - e^{-(t_s - t_a)}\right) E\left[\left(f_4^{(\tau)}((X,2); t \le t_s)\right)^2\right] + \frac{2e^{-(t_s - t_a)}}{3} E\left[\left(f_4^{(\tau)}((1,2); t > t_s)\right)^2\right], \tag{13}$$

where we combined terms by using that all lineages are exchangable at $t > t_s$ and therefore $E\left[\left(f_4^{(\tau)}((X,2); t > t_s)\right)^2\right] = E\left[\left(f_4^{(\tau)}((1,2); t > t_s)\right)^2\right]$.

2.4 Evaluating the theoretical z-score given a *Twigstats* cutoff

We can now substitute Eqs. (7) and (11) in Eq. (10). If we can evaluate the three integrals given by

- $E\left[f_4^{(\tau)}((X,2); t \le t_s)\right]$
- $E\left[\left(f_4^{(\tau)}((X,2); t \le t_s)\right)^2\right]$
- $E\left[\left(f_4^{(\tau)}((1,2); t > t_s)\right)^2\right]$

then we can compute a theoretical z-score as a function of the *Twigstats* cutoff time and search for the optimal value. We implemented a function `theoretical_zscore` computing this theoretical z-score in our *Twigstats* package. Arguments to this function are the cutoff time (in generations), admixture date, source split time, and admixture proportion.

To evaluate the integrals, we start with $E\left[f_4^{(\tau)}((X,2); t \le t_s)\right]$. We need to integrate over times when lineages X and 2 coalesce, and then, if $\tau > t_s$, also over times when the coalesced lineage coalesces with 1. We obtain

$$E\left[f_4^{(\tau)}((X,2); t \le t_s)\right] = \frac{-1}{1 - e^{-(t_s - t_a)}}\left[1_{\{\tau > t_s\}} \int_{t_a}^{t_s} \int_{t_s}^{\tau} (t_2 - t_1) e^{-(t_1 - t_a)} e^{-(t_2 - t_s)} dt_2\right. \tag{14}$$

$$+ (\tau - t_1) e^{-(t_1 - t_a)} e^{-(\tau - t_s)} dt_1 \tag{15}$$

$$\left. + 1_{\{\tau \le t_s\}} \int_{t_a}^{\tau} (\tau - t_1) e^{-(t_1 - t_a)} dt_1\right]. \tag{16}$$

The second moment $E\left[\left(f_4^{(\tau)}((X,2); t \le t_s)\right)^2\right]$ takes a similar form and we obtain

$$E\left[\left(f_4^{(\tau)}((X,2); t \le t_s)\right)^2\right] = \frac{1}{1 - e^{-(t_s - t_a)}}\left[1_{\{\tau > t_s\}} \int_{t_a}^{t_s} \int_{t_s}^{\tau} (t_2 - t_1)^2 e^{-(t_1 - t_a)} e^{-(t_2 - t_s)} dt_2\right. \tag{17}$$

$$+ (\tau - t_1) e^{-(t_1 - t_a)} e^{-(\tau - t_s)} dt_1 \tag{18}$$

$$\left. + 1_{\{\tau \le t_s\}} \int_{t_a}^{\tau} (\tau - t_1)^2 e^{-(t_1 - t_a)} dt_1\right]. \tag{19}$$

Finally, the second moment $E\left[\left(f_4^{(\tau)}((1,2); t > t_s)\right)^2\right]$ equals 0 if $\tau \le t_s$ and so is given by

$$E\left[\left(f_4^{(\tau)}((1,2); t \le t_s)\right)^2\right] = 1_{\{\tau > t_s\}}\left[\int_{t_s}^{\tau} \int_{t_1}^{\tau} (t_2 - t_1)^2 3e^{-3(t_1 - t_s)} e^{-(t_2 - t_1)} dt_2 dt_1\right. \tag{20}$$

$$\left. + \int_{t_s}^{\tau} (\tau - t_1)^2 3e^{-3(t_1 - t_s)} e^{-(\tau - t_1)} dt_1\right] \tag{21}$$

These integrals are straight-forward to compute using integration by parts, which is implemented in the `theoretical_zscore` function in our *Twigstats* package (`https://github.com/leospeidel/twigstats`).

2.5 Evaluating the optimal *Twigstats* cutoff

We use our function `theoretical_zscore` that computes the theoretical z-score of Eq. (9) to evaluate how the optimal *Twigstats* cutoff is impacted by admixture date, source split time, and admixture proportion.

The first panel in Extended Data Figure 3a shows how the theoretical z-score varies as a function of the admixture proportion. In the second panel, we observe that a younger source split time will lead to a larger fold-increase in the optimal z-score, confirming our hypothesis that the *Twigstats* ascertaiment scheme is particularly beneficial with closely related source groups. The third panel shows that the optimal *Twigstats* cutoff is greater than the source split time and scales with both the admixture proportion and source split time. The fourth panel shows that the optimal cutoff appears to be bounded by by a factor of less than 1.4 times the source split time. We observe a good agreement with the optimal *Twigstats* cutoff observed when simulating data (Extended Data Fig. 3b.)

References

[1] Robert Maier et al. "On the limits of fitting complex models of population history to f-statistics". In: *Elife* 12 (Apr. 2023), e85492.
