# Supplementary material for: High-resolution genomic history of early medieval Europe
Source: Nature. 2025 Jan 1;637(8044):118–26. doi: 10.1038/s41586-024-08275-2 (PMC11693606; doi:10.1038/s41586-024-08275-2)
Supplement: Supplementary file 2 — Reporting Summary [file 41586_2024_8275_MOESM2_ESM.pdf]

nature portfolio

Corresponding author(s): Leo Speidel, Pontus Skoglund

Last updated by author(s): 24/9/24

Reporting Summary

Nature Portfolio wishes to improve the reproducibility of the work that we publish. This form provides structure for consistency and transparency in reporting. For further information on Nature Portfolio policies, see our Editorial Policies and the Editorial Policy Checklist.

Statistics

For all statistical analyses, confirm that the following items are present in the figure legend, table legend, main text, or Methods section.

n/a	Confirmed	
☐	☒	The exact sample size (n) for each experimental group/condition, given as a discrete number and unit of measurement
☐	☒	A statement on whether measurements were taken from distinct samples or whether the same sample was measured repeatedly
☐	☒	The statistical test(s) used AND whether they are one- or two-sided<br>*Only common tests should be described solely by name; describe more complex techniques in the Methods section.*
☐	☒	A description of all covariates tested
☐	☒	A description of any assumptions or corrections, such as tests of normality and adjustment for multiple comparisons
☐	☒	A full description of the statistical parameters including central tendency (e.g. means) or other basic estimates (e.g. regression coefficient) AND variation (e.g. standard deviation) or associated estimates of uncertainty (e.g. confidence intervals)
☐	☒	For null hypothesis testing, the test statistic (e.g. F, t, r) with confidence intervals, effect sizes, degrees of freedom and P value noted<br>*Give P values as exact values whenever suitable.*
☐	☒	For Bayesian analysis, information on the choice of priors and Markov chain Monte Carlo settings
☐	☒	For hierarchical and complex designs, identification of the appropriate level for tests and full reporting of outcomes
☐	☒	Estimates of effect sizes (e.g. Cohen's d, Pearson's r), indicating how they were calculated

Our web collection on statistics for biologists contains articles on many of the points above.

Software and code

Policy information about availability of computer code

Data collection	No software was used for data collection.
Data analysis	We used bcftools 1.19, samtools 1.3.1, bwa aln 0.7.17-r1188, GLIMPSEv1.1.1, Relate v1.2.1, and R packages stats (v3.6.2), admixtools2 (v2.0.4). Code for twigstats (v1.0.1) is available through https://github.com/leospeidel/twigstats and https://zenodo.org/records/13833120.

For manuscripts utilizing custom algorithms or software that are central to the research but not yet described in published literature, software must be made available to editors and reviewers. We strongly encourage code deposition in a community repository (e.g. GitHub). See the Nature Portfolio guidelines for submitting code & software for further information.

Data

Policy information about availability of data

All manuscripts must include a data availability statement. This statement should provide the following information, where applicable:

- Accession codes, unique identifiers, or web links for publicly available datasets
- A description of any restrictions on data availability
- For clinical datasets or third party data, please ensure that the statement adheres to our policy

All ancient DNA data used in this study was publically available and is listed in Extended Data Table 1. The corresponding accession codes are: ERS2540893, PRJEB11004, PRJEB11364, PRJEB11848, PRJEB11995, PRJEB13123, PRJEB14180, PRJEB14675, PRJEB14737, PRJEB18067, PRJEB20614, PRJEB20658, PRJEB21037, PRJEB21330, PRJEB21940, PRJEB22592, PRJEB23467, PRJEB26760, PRJEB29189, PRJEB29360, PRJEB29360 , PRJEB29603, PRJEB29700, PRJEB31045, PRJEB31249,

PRJEB31893, PRJEB32566, PRJEB32786, PRJEB33128, PRJEB34091, PRJEB35327, PRJEB36854, PRJEB37976, PRJEB39316, PRJEB40698, PRJEB41240, PRJEB4604, PRJEB46830, PRJEB48333, PRJEB49419, PRJEB49524, PRJEB49971, PRJEB50857, PRJEB53565, PRJEB53670, PRJEB54899, PRJEB57364, PRJEB59976, PRJEB6090, PRJEB6272, PRJEB64656, PRJEB65098, PRJEB65239, PRJEB6622, PRJEB6915, PRJEB7618, PRJEB9021, PRJNA230689, PRJNA280812, PRJNA812074, SRP029640, SRP039766, SRP132581

Research involving human participants, their data, or biological material

Policy information about studies with human participants or human data. See also policy information about sex, gender (identity/presentation), and sexual orientation and race, ethnicity and racism.

Reporting on sex and gender	We use DNA sequenced from archaeological remains and have inferred the genetic sex where possible.
Reporting on race, ethnicity, or other socially relevant groupings	We have grouped ancient DNA samples by expert assigned archaeological context, by time period, by geographic location and by genetic clustering.
Population characteristics	We have included samples from Western and Central Eurasia spanning the last 10,000 years.
Recruitment	We used publicly available ancient DNA samples. These are subject to sampling bias, that may arise for instance due to burial context. In particular, current technologies are unable to extract DNA from cremation burials which have been frequent in some cultural contexts.
Ethics oversight	N/A

Note that full information on the approval of the study protocol must also be provided in the manuscript.

Field-specific reporting

Please select the one below that is the best fit for your research. If you are not sure, read the appropriate sections before making your selection.

☒ Life sciences ☐ Behavioural & social sciences ☐ Ecological, evolutionary & environmental sciences

For a reference copy of the document with all sections, see nature.com/documents/nr-reporting-summary-flat.pdf

Life sciences study design

All studies must disclose on these points even when the disclosure is negative.

Sample size	We aimed to compile a close to exhaustive list of ancient genomes with Western and Central Eurasian ancestries and then filtered by sequencing technology (shotgun sequencing), sequencing coverage (>0.5x), and excluded close relatives. Our final dataset comprised 1,151 genomes in total.
Data exclusions	We only used samples that were sequenced genome-wide to an average sequencing coverage of 0.5x. We excluded close relatives.
Replication	We conducted two replication analyses of previous work (Extended Data Figure 8) to make sure our findings are consistent with current knowledge. We conducted non-parametric and parametric modeling to confirm that our findings are robust to some modeling assumptions.
Randomization	We ran ancestry models both on a per individual basis, as well as grouping individuals according to archaeological context provided by the reference and as detailed in SI Table 1. To select source groups in our ancestry modelling, we used a clustering approach described in the Methods section.
Blinding	We used existing data and so blinding was not possible.

Reporting for specific materials, systems and methods

We require information from authors about some types of materials, experimental systems and methods used in many studies. Here, indicate whether each material, system or method listed is relevant to your study. If you are not sure if a list item applies to your research, read the appropriate section before selecting a response.

Materials & experimental systems

n/a	Involved in the study
☒	☐ Antibodies
☒	☐ Eukaryotic cell lines
☒	☐ Palaeontology and archaeology
☒	☐ Animals and other organisms
☒	☐ Clinical data
☒	☐ Dual use research of concern
☒	☐ Plants

Methods

n/a	Involved in the study
☒	☐ ChIP-seq
☒	☐ Flow cytometry
☒	☐ MRI-based neuroimaging

Plants

Seed stocks

Report on the source of all seed stocks or other plant material used. If applicable, state the seed stock centre and catalogue number. If plant specimens were collected from the field, describe the collection location, date and sampling procedures.

Novel plant genotypes

Describe the methods by which all novel plant genotypes were produced. This includes those generated by transgenic approaches, gene editing, chemical/radiation-based mutagenesis and hybridization. For transgenic lines, describe the transformation method, the number of independent lines analyzed and the generation upon which experiments were performed. For gene-edited lines, describe the editor used, the endogenous sequence targeted for editing, the targeting guide RNA sequence (if applicable) and how the editor was applied.

Authentication

Describe any authentication procedures for each seed stock used or novel genotype generated. Describe any experiments used to assess the effect of a mutation and, where applicable, how potential secondary effects (e.g. second site T-DNA insertions, mosiacism, off-target gene editing) were examined.
